# Testing for Faecal Gluten Immunogenic Peptides: Is It Useful to Evaluate Adherence to Gluten‐Free Diet?

**DOI:** 10.1111/apa.70187

**Published:** 2025-06-20

**Authors:** Sabrina Cenni, Marianna Casertano, Elisabetta D'Addio, Daniela Pacella, Carlo Tolone, Massimo Martinelli, Annamaria Staiano, Erasmo Miele, Caterina Strisciuglio

**Affiliations:** ^1^ Department of Experimental Medicine University of Campania ‘Luigi Vanvitelli’ Naples Italy; ^2^ Department of Woman, Child and General and Specialist Surgery University of Campania ‘Luigi Vanvitelli’ Naples Italy; ^3^ Department of Public Health University of Naples ‘Federico II’ Naples Italy; ^4^ Department of Translational Medical Science, Section of Pediatrics University of Naples ‘Federico II’ Naples Italy

**Keywords:** Biagi score, celiac disease (CD), faecal gluten immunogenic peptides, gluten free diet

## Abstract

**Aim:**

Determination of faecal gluten immunogenic peptides (f‐GIP) has recently been proposed as new noninvasive method to detect gluten intake in celiac disease (CD). Our aim was to evaluate the use of f‐GIP for the adherence to gluten‐free diet (GFD) and to compare this new marker with traditional methods.

**Methods:**

We enrolled both newly diagnosed cases and children in follow‐up with CD. Adherence to the GFD was assessed using the Biagi score, IgA anti‐transglutaminase (tTG IgA) and f‐GIP.

**Results:**

Seventy‐one CD children, 40.8% new diagnosis and 59.2% in follow‐up, were enrolled. Significant differences were found in the levels of faecal GIP between new diagnosis and follow up (median 185.7 ng/mL, IQR 67.8–318.8 ng/mL and 16.6 ng/mL, IQR 6.9–61 ng/mL respectively, *p* < 0.001). A significant direct correlation was found between faecal GIP and tTG IgA in the total cohort of enrolled patients (*r* = 0.5, *p* < 0.001). In the follow up group, 21.4% tested positive for GIP and, of these GIP + patients, 78% had a high Biagi score.

**Conclusion:**

Our data shows that faecal GIP offers a precise assessment of even occasional exposure to gluten and suggests it might represent a less invasive instrument to check dietary adherence compared to traditional methods.

**Abbreviations**
ALT: alanine aminotransferase
AST: aspartate amino transferase
CD: celiac disease
ELISA: enzyme‐linked immunosorbent assay
ESPGHAN: European Society for Paediatric Gastroenterology Hepatology and Nutrition
GFD: gluten‐free diet
GIP: faecal gluten immunogenic peptides
Hb: haemoglobin
Ht: haematocrit
MCV: Mean Corpuscular Volume
tTG IgA: anti‐transglutaminase antibodies

**Summary**

- Ensuring compliance with a gluten‐free diet is crucial for managing coeliac disease (CD).
- We demonstrated that faecal gluten immunogenic peptides (GIPs) offer a non‐invasive method to detect gluten intake, providing a precise assessment of occasional exposure compared to traditional methods.
- Faecal GIP It helps identify non‐adhering patients and can be used in refractory CD as an additional tool to decide whether an endoscopy is needed.

## 1 Introduction

Celiac disease (CD) is the most common chronic gastrointestinal disorder in Western countries. Currently, the only effective treatment is strict adherence to a lifelong gluten‐free diet (GFD) [1]. However, following a GFD consistently is challenging. In fact, at least one third of patients with CD report not fully adhering to the GFD. Several studies have shown that between 30% and 75% of celiac patients fail to follow the GFD [2, 3, 4, 5]. Moreover, 36% to 55% of patients who claim to adhere completely to a GFD do not achieve histological remission, probably due to the frequent ‘accidental’ gluten ingestion [6]. Adolescents are more likely to struggle with GFD adherence than younger children [7]. Adherence assessment of GFD is based on clinical evaluations, serological tests with anti‐transglutaminase antibodies (tTG IgA), demonstration of mucosal healing through duodenal biopsy and structured questionnaires that investigate the quality and frequency of foods consumed assessed by expert dietitians [8]. However, none of these methods provide an entirely accurate measure of patient adherence to the GFD [9]. The Biagi questionnaire remains the most widely used and quickest tool, consisting of four simple questions that generate a score with proven reliability, correlating well with histology [9, 10]. However, questionnaires rely on subjective patient responses, which may underestimate gluten intake, overlook accidental consumption or omit information intentionally. Additionally, while the Biagi questionnaire has been used in paediatric studies, it has not yet been validated for use in children. Regarding antibodies, recent studies have shown that normalisation of antibodies does not always correlate with mucosal healing [11]. In fact, it has been observed that some patients who claim to follow a gluten‐free diet and test negative for antibodies still exhibit intestinal mucosal damage on biopsy, indicating gluten exposure [11]. Histological evaluation remains the best marker of gluten exposure, but it requires an invasive, risky and expensive procedure that is not always well accepted, particularly by asymptomatic patients. For these reasons, we have to find more reliable markers that allow us to evaluate gluten exposure in a less invasive way and that correlate well with actual mucosal healing. Tests for detection of gluten immunogenic peptides (GIP) are a recently introduced method that allows early identification of small amounts of gluten consumption [12, 13, 14]. These peptides are small protein fragments that resist digestion and are partly excreted in the faeces, accounting for most of the immunotoxic reactions to gluten. They interact with the immune system of patients with CD, triggering a response against various antigens [15]. Preliminary studies have shown that GIP tests have a sensitivity of 98.5% and a specificity of 100% [16]. For this reason, detection of faecal GIP has been proposed as an effective, noninvasive method of assessing gluten exposure. The aim of this study was to confirm that measuring GIP in faeces can be used as an indicator of adherence to a GFD and to compare this new marker with traditional methods for assessing gluten exposure.

This is a small‐scale study that confirms paediatric age findings from similar research conducted in the adult population.

## 2 Material and Methods

We conducted an observational cross‐sectional study between October 2019 and May 2021. The study included patients with CD diagnosed based on the criteria of the European Society for Paediatric Gastroenterology Hepatology and Nutrition (ESPGHAN) [17] both newly diagnosed and those in follow up, and managed at the Paediatric Gastroenterology Unit of University of Campania ‘Luigi Vanvitelli’. We collected stool samples from newly diagnosed patients before they started a GFD. The follow up group consisted of CD patients following a GFD for at least 2 years prior to study inclusion. Exclusion criteria included the history of kidney or liver disease, suspected other gastrointestinal conditions, use of medications. We also excluded patients diagnosed with non‐celiac gluten sensitivity, those with Marsh 1 lesions on duodenal biopsy and patients lost to follow‐up. Additionally, patients unable to provide informed consent were excluded. At the time of enrollment, baseline patients' characteristics were extracted from medical records: age, sex, disease duration. Adherence to the GFD was assessed using the Biagi score, based on a structured questionnaire completed with the help of an expert dietitian, along with the level of tTG IgA and evaluation of faecal GIP. We instructed the subjects not to change their dietary habits in the days preceding the collection of stool samples in order not to alter the dosage of GIP. Parents, legal guardians or children over 12 years old in follow up completed the Biagi questionnaire with the dietitian's assistance at the time of the stool and blood sample collection. A score of 0 or 1 indicates that the patient is not on a strict GFD, a score of 2 indicates that the patient follows the diet but makes mistakes that need to be corrected, and scores of 3 or 4 indicate that the patient follows the diet strictly [10].

### 2.1 Sample Collection and Preparation

Blood samples were taken from each patient at 8:00 AM after an overnight fast to determine serum markers of nutritional status haemoglobin (Hb), haematocrit (Ht), Mean Corpuscular Volume (MCV), albumin, AST: aspartate amino transferase; ALT: alanine aminotransferase and tTG IgA. The quantitative determination of serum tTG IgAwas achieved by means of a sandwich type enzyme immunoassay according to manufacture instructions (Eurospital, Italy). Titers of ≤ 15 U/mL were considered negative. Faecal samples were provided by patients. Samples were stored at −80°Cat Department of Paediatrics, University of Campania ‘Luigi Vanvitelli’, Naples, Italy. Faecal samples were analysed at Immundiagnostik AG Center, Bensheim, Germany to determine the GIP levels. GIP levels were measured by enzyme‐linked immunosorbent assay (ELISA) following the manufacturer's instructions, with the kit of Immundiagnostik AG (IDK GIP‐ELISA: upper and lower limit of quantification is between 0.078 and 1.25 μg GIP/g (78 and 1250 ng/mL) stool). This test is a sandwich ELISA assay designed to detect and quantify gluten immunogenic peptides (essentially peptides related to the 33‐mer peptide) in stool samples.

### 2.2 Statistical Analysis

Data are presented as frequency (percentages) for the categorical variables, while they are presented as mean ± standard deviation for continuous variables that are normally distributed, or as median and interquartile range for non‐parametric distributions. Shapiro–Wilk test was used to assess normality. Student's t test for independent samples or Mann–Whitney *U* as appropriate were used to analyse the differences between groups for quantitative variable. Correlation was measured with the Pearson's correlation coefficient or with the Spearman's correlation coefficient as appropriate. The confusion matrix for the descriptive diagnostic performance analyses was constructed considering the GIP levels < 78 ng/mL as negative and Ab anti tTG IgA < 15 U/mL as negative. For all analyses, a *p*‐value < 0.05 was considered statistically significant. All analyses were performed using R statistical software version 4.0.3. Concordance is measured using raw agreement and Cohen's kappa, which can be interpreted as follows: < 0.20, poor concordance; 0.20–0.40, fair concordance; 0.41–0.60, moderate concordance; 0.61–0.80, good concordance; > 0.81, very good concordance.

### 2.3 Ethical Considerations

The Institutional Review Board of the University of Campania ‘Luigi Vanvitelli’ approved the study protocol with the registration number 207/20. Informed consent was obtained from the parents of all children and from the children themselves if older than 12 years.

## 3 Results

**FIGURE 1:**
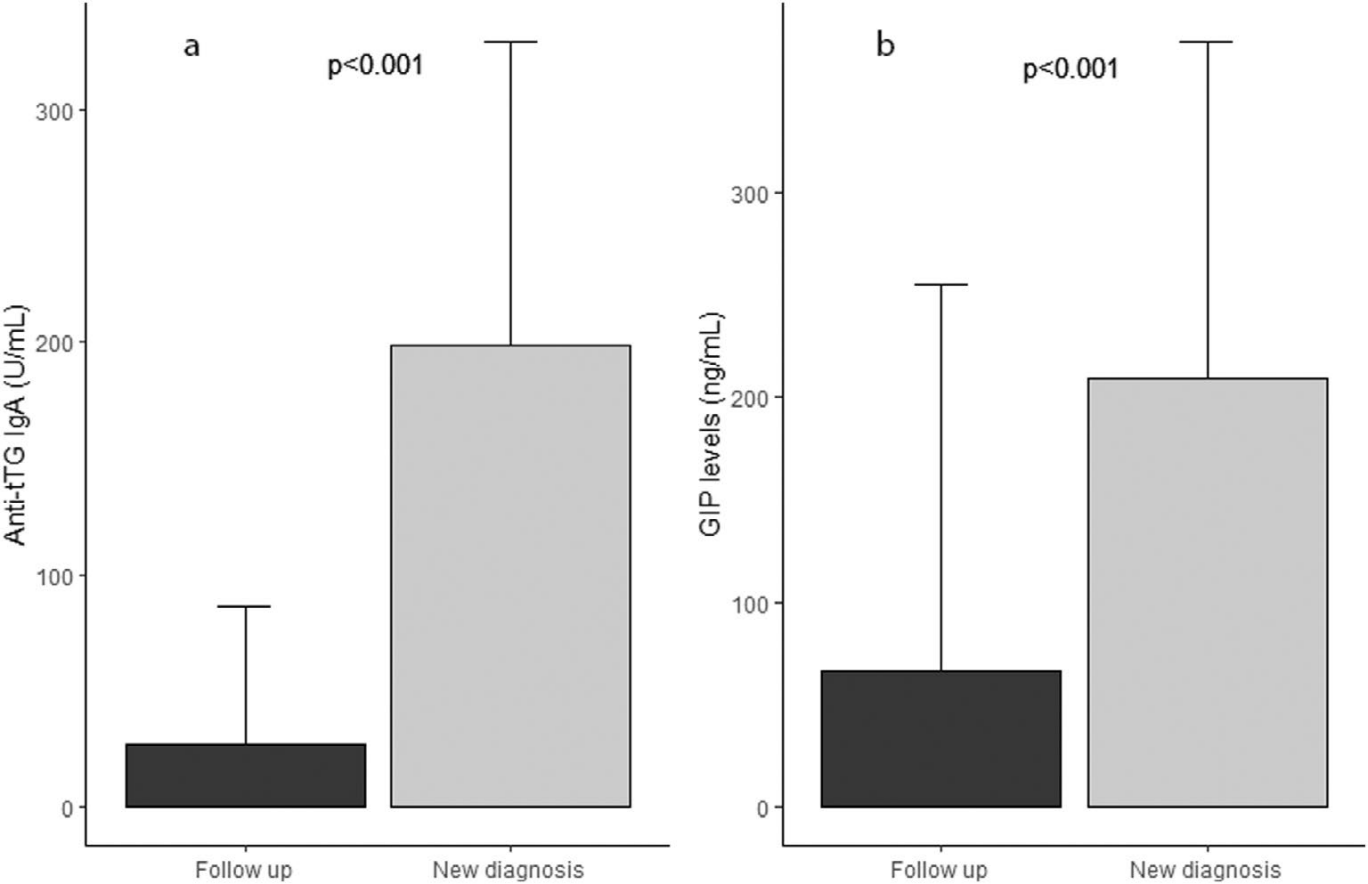
(a) Comparison of anti‐tTG IgA levels between newly diagnosed and follow‐up patients. (b) Comparison of faecal GIP levels between newly diagnosed and follow‐up patients.

**FIGURE 2:**
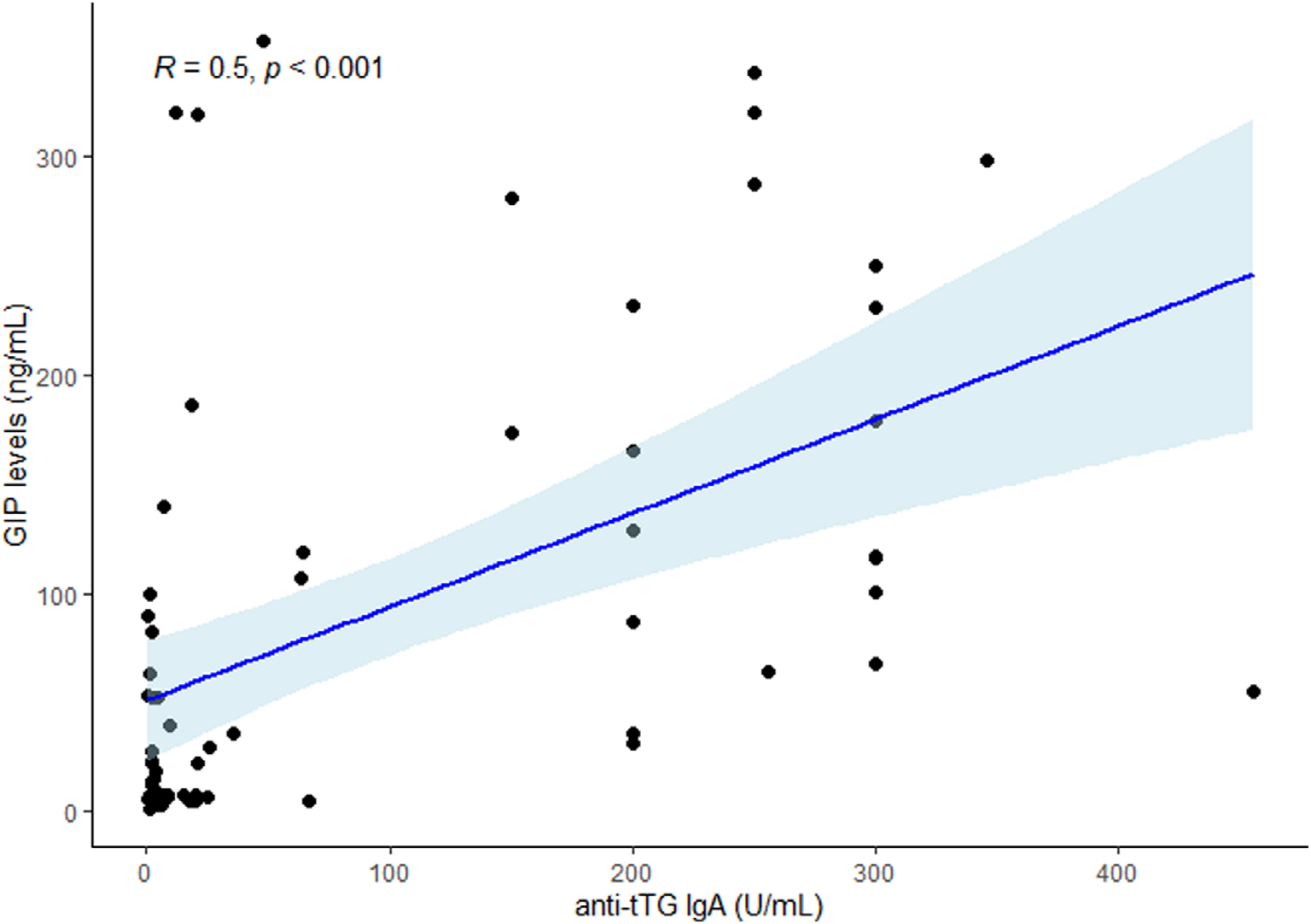
Correlation between faecal GIP and tTG IgA in all CD children. CD, celiac disease; GIP, Gluten immunogenic peptides; tTG IgA, anti‐transglutaminase antibodies.

The final sample included 71 patients (47.8% male, 52.2% female), 29 (40.8%) newly diagnosed and 42 (59.2%) in follow up with a mean age at time of enrollment of 9.1 ± 4.9 years. CD children in follow up had been on a GFD for a mean 4.71 ± 2.6 years. Children characteristics are presented in Table 1. Anti‐tTG IgA values were elevated at diagnosis in all patients compared to follow‐up patients (median tTG IgA 200 U/mL, IQR 64–300 U/mL vs. median 4.25 U/mL, IQR 2–17.35 U/mL, respectively; *p* < 0.001) (Figure 1a). Accordingly, we found significant differences in the levels of faecal GIP between new diagnosis and follow up CD children (median 185.7 ng/mL, IQR 67.8–318.8 ng/mL and 16.6 ng/mL, IQR 6.9–61 ng/mL respectively, *p* < 0.001) (Figure 1b). After removing outliers, moderate and significant direct correlation was found between faecal GIPs and tTG IgA in the total cohort of enrolled patients (*r* = 0.5, *p* < 0.001) (Figure 2). There was no correlation between serum markers of nutritional status and both tTG IgA and GIPs levels.

**TABLE 1:** Clinical and laboratory features of CD children included in the study.

	Newly diagnosed, *N* = 29	Previously diagnosed, *n* = 42
Sex, males (%)	11 (38)	23 (54)
Age, years, mean ± SD	6.17 ± 3.5	10.9 ± 4.7
Duration of disease, years, mean ± SD	NA	4.71 ± 2.6
Marsh‐Oberhuber at diagnosis, *n* (%)		
Marsh‐Oberhuber 3	5 (17.2)	22 (52.3)
Marsh‐Oberhuber 2	4 (13.8)	4 (9.5)
Diagnosing CD without performing EGDS according to ESPGHAN guidelines	20 (69)	15 (35.7)
tTG IgA U/mL, median [IQR]	200 [64–300]	4.25 [2–17.35]
Faecal GIP ng/mL median [IQR]	185.7 [67.8–318.8]	16.6 [6.9–61]

Abbreviations: CD, celiac disease; EGDS, esophagogastroduodenoscopy; GIP, gluten immunogenic peptides; IQR, interquartile range; SD, standard deviation; tTG IgA, anti‐transglutaminase antibodies.

### 3.1 Biagi Score and tTG IgA Compared With Faecal Gluten Immunogenic Peptide Levels

In the follow up group nine patients (21.4%) tested positive for faecal GIP (GIP+) and 33 patients (78.6%) tested negative (GIP−). In positive subjects, GIP values ranged from 82.6 to 1224.7 ng/mL. Between GIP+ group and GIP− group no difference was found in age, gender or endoscopic features. Performance of the Biagi score was evaluated in all children with CD in follow up on recommendation to adhere to a GFD. Biagi score was evaluated as a continuous variable. We also dichotomized the Biagi score and divided patients into two groups: patients not on a GFD or who make mistakes (score 0–2) and patients with a good adherence to the GFD (score 3–4). In our study, 90.5% (38/42) of patients adhered to the GFD based on the Biagi score, however 21.4% (9/42) had high levels of faecal GIP. Of the 38 patients with declared good adherence, 78% had a positive GIP test. Thirteen of 42 patients (30.9%) had elevated IgA tTG (median value 200 U/mL). We found no differences in this group of patients in terms of age, gender, duration of GFD and IgA tTG level compared to patients with good GFD adherence and negative GIP.

On the other hand, of the four patients who admitted poor adherence, two had negative GIP tests. According to the Biagi score, 31/33 (94%) of GIP− patients showed strict dietary adherence, whereas 6.1% (2/33) of GIP− made substantial mistakes. In the group of patients with GIP+, 22% (2/9) were making substantial mistakes, showing a Biagi score of 0–2. No correlation was found between GIP levels and Biagi score. Raw agreement between GIP and Biagi score is equal to 78.6%, while concordance is fair and is equal to *k* = 0.20 (95% CI 0; 0.54).

When it came to anti‐tTG IgA, 9 of 42 (21.4%) patients in follow up had positive tTG IgA antibodies. Anti‐tTG IgA were found in 44% GIP+ patients and in 27% GIP− patients. In the latter group, however, antibody values were all > 1.5 times the cutoff value. Raw agreement between GIP and anti‐tTG is equal to 66.7%, while concordance is poor and is equal to *k* = 0.15 (95% CI 0; 0.46). Comparison between the two groups of GIP positive and negative for the prevalence of positivity of Biagi score and TTG IgA persistence and other clinical variables are reported in Table 2.

**TABLE 2:** Comparison between the two groups of GIP positive and negative for the prevalence of positivity of Biagi score and TTG IgA persistence and other clinical variables.

	GIP negative, *N* = 33	GIP positive, *N* = 9	*p*
Sex, males (%)	18 (55%)	5 (56%)	> 0.999
Age, years, mean ± SD	11.3 **±** 4.6	9.8 **±** 5.2	0.442
Marsh‐Oberhuber at diagnosis, *n* (%)			
2	4 (19%)	0 (0%)	0.555
3	17 (81%)	5 (100%)	
Biagi score, *n* (%)			
Negative	31 (94%)	7 (78%)	0.196
Positive	2 (6.1%)	2 (22%)	
Anti‐tTG, *n* (%)			
Negative	24 (73%)	5 (56%)	0.422
Positive	9 (27%)	4 (44%)	

Abbreviations: GIPs, gluten immunogenic peptides; tTG IgA, anti‐transglutaminase antibodies.

## 4 Discussion

In this study, we assessed the role of faecal GIP in evaluating adherence to a GFD compared to serological tests and the Biagi questionnaire in a paediatric cohort of CD children. We found that GIP testing revealed the lowest adherence rate to the GFD. As expected, high levels of faecal GIP were detectable in patients with newly diagnosed CD (indicating high gluten exposure) compared to follow‐up patients on GFD. Additionally, we found a positive correlation between faecal GIP levels and tTG IgA, which is currently the most standardised method for monitoring paediatric celiac patients according to the recent ESPGHAN position paper [18].

The current routine approach to evaluating dietary a includes a comprehensive assessment of CD patients, involving food history, structured questionnaires, clinical symptoms, tTG IgA antibody assays and duodenal biopsy, which is reserved for the most difficult and refractory cases [19, 20]. The choice of the method for monitoring gluten exposure must be based on accuracy, invasiveness, time and cost of each, taking into account the limitations of the available options. Clinical improvement usually reflects good adherence, but it cannot be applied to patients identified through screening who are asymptomatic at the time of diagnosis [20].

Questionnaires, especially the Biagi score, are useful and easy to perform. They are a non‐invasive, well tolerated by patients, particularly in paediatric populations, but they are subjective and measure the patient's intention to follow GFD rather than actual gluten exposure [10]. On the other hand, serological testing which involves a blood sample, is widely used for routine monitoring of CD patients on GFD to measure chronic gluten exposure, although the results do not always correlate correlated with histological findings, symptoms and dietary adherence [15, 21]. In some cases, antibody levels may not normalise despite adherence to the diet due to their long half‐life and because they reflect immune activity rather than mucosal damage [1]. This could be due to the long half‐life of antibodies and to the fact that antibody levels reflect immune response rather than direct intestinal damage.

The measurement of GIP in stool or urine has been introduced as a tool to detect gluten ingestion in patients on a GFD [15, 16, 22, 23, 24]. Detection of GIP indicates accidental exposure or voluntary lapses in diet adherenc. The study of Comino et al. [14] demonstrated that gluten‐derived peptides can be sensitively detected in human faeces with a positive correlation to the amount of gluten ingested between Days 2 and 4 after consumption. Therefore, this method is very useful to evaluate diagnosis of gluten ingestion or evaluating acute CD symptoms in individuals already on a GFD. However, a limitation of faecal GIP is that it does not measure chronic exposure like antibodies. Furthermore, recent study confirmed a weak association between the amount of gluten ingested and faecal GIP excretion and detecting sporadic gluten exposure may require multiple stool samples to achieve a sensitivity above 90% [25].

Compared to other methods for evaluating gluten exposure, we found that GIP testing revealed the lowest adherence rate to the GFD (75%), suggesting that this noninvasive assay should be more sensitive than others in detecting occasional exposition to inadvertent gluten [26, 27].

In our study, 90.5% (38/42) of patients adhered to the GFD based on the Biagi score, however 21.4% of the celiac patients on a GFDhad detectable amounts of GIP in their stools, indicating non‐compliance. Of these GIP‐positive patients, 78% claimed they had not consumed any gluten, and were therefore not identified by the questionnaire and only 22% had a low Biagi score.

In contrast, gluten exposure was detected in 9.5% of patients when assessing adherence by Biagi questionnaire and in 28.5% of the children by using anti‐tTG antibodies.

Similar results were reported in a study by Comino et al. [16], which assessed GFD adherence in 138 children and adult celiac patients using both faecal GIP and food questionnaires. In that study, 28.3% had positive GIP test, while only 18.1% were non‐compliant according to the food questionnaire. Among patients with a positive GIP test, 69.2% reported good adherence to the GFD based on the questionnaire, while 23.1% were non‐compliant by both methods [16]. Unlike the study by Comino et al., we found no correlation between faecal GIP levels and age, gender or disease duration.

The paediatric study by Gerasimidis et al. showed that faecal GIP was detectable in 16% of patients with CD diagnosis on GFD. Compared to GIP, the Biagi score and tTG, presented sensitivity of 17% and 42%, respectively [28]. The authors concluded that while tTG IgA and the Biagi score had high sensitivity for identifying patients who did not consume gluten, their sensitivity for detecting all patients who consumed gluten was relatively low [28].

Our results confirm the limitations of questionnaires: since they are based on patient perceptions rather than objective data, they cannot identify voluntary or accidental dietary transgressions. Patients may intentionally omit gluten intake, may not know the exact gluten content of what they consume, or may unknowingly eat foods containing gluten. Several studies have previously used this test to estimate gluten exposure, typically finding adherence rates between 70.2% and 84% [16, 28, 29]. Serological testing is commonly used in the routine monitoring of CD patients on GFD, although the results are not always correlated with histological findings or symptoms [21]. Additionally, serological tests may fail to detect occasional gluten exposure [30]. In our study, 21.4% of CD children had positive tTG IgA antibody levels. These values were likely not due to antibody persistence, as the subjects had been on a GFD for at least 2 years. Concentrations of tTG IgA > 15 U/mLwere only found in only 44% of GIP+ patients. More than 50% of patients with a positive GIP test showed negative anti‐tTG IgA antibody levels, a finding similar to that reported in previous studies [31]. This may be due to occasional gluten exposure, which is poorly detected by serum anti‐tTG IgA testing. These results are in line with data in the literature showing the low sensitivity of these serum markers in monitoring dietary adherence [20], and most importantly, indicate that they are not effective in detecting small or infrequent gluten exposure [32].

Detection of GIP helps identify gluten intake or transgressions earlier than other methods, such as serological testing [22, 27, 28]. It is also important to consider the role of deamidated gliadin peptide (DGP) antibodies which have been shown in previous studies to be sensitive markers of dietary transgressions. Several studies have demonstrated that DGP antibodies, can remain elevated after recent gluten exposure, often before changes appear in tTG‐IgA levels and their presence correlates with mucosal damage and can signal dietary non‐compliance, although the window of detection is longer and less precise than GIP. Stefanolo et al. in a recent observational study investigate the efficacy of weekly stool GIP measurements compared to conventional methods—such as self‐assessment questionnaires, CD serology and expert dietary evaluations for assessing adherence to a GFD in patients with CD. Over 4 weeks, 53 adult CD patients provided weekly stool samples for GIP analysis. The findings revealed that patients with higher stool GIP concentrations exhibited elevated serum levels of IgA deamidated gliadin peptides (DGPs). Like our data, the study concluded that stool GIP measurements offer a valuable and practical tool for monitoring GFD adherence, potentially identifying gluten exposure not detected by traditional assessment methods [33]. Utilising both markers allows for a comprehensive assessment, identifying instances of gluten ingestion and evaluating the corresponding immune reactions, thereby improving the management of CD.

Moreover, it has been demonstrated that GIP assays are highly accurate in identifying patients with persistent intestinal atrophy [15, 16, 34]. The ideal frequency of GIP determinations depends on the purpose of testing and the patient's clinical situation. Since GIP reflects recent gluten exposure (within 2–4 days), testing strategy should be tailored to capture potential intermittent or low‐level gluten intake. General recommendations on when to perform GIP tests could be: weekly testing (for 4–6 weeks during the initial phase of monitoring or when symptoms persist), monthly testing (for long‐term monitoring or during follow‐up) and random spot checks useful in cases where gluten exposure is suspected despite a lack of symptoms [28, 34].

Repeated stool GIP testing could offer a more comprehensive assessment of the patient's overall adherence to dietary recommendations over time. Several studies confirm stool GIP levels were highly sensitive to gluten consumption, with levels correlating with the amount of gluten ingested and the time elapsed since ingestion [14]. Regular monitoring would help to detect trends or patterns of non‐adherence that may not be captured by a single test. A single test could miss such intermittent exposures, especially since GIP is detectable in stool only for a limited time after gluten ingestion (roughly 2–4 days). Repeated testing increases the likelihood of catching accidental or low‐level gluten exposure. While guidelines are still evolving, experts generally recommend serial testing (e.g., weekly or monthly for a period) if: there are symptoms despite a gluten‐free diet, there's uncertainty about dietary adherence or to the effectiveness of diet counselling [35].

In conclusion, a single stool GIP test might provide some immediate insight into recent dietary indiscretions, but repeated testing offers a completer and more accurate picture of dietary adherence, helping to prevent long‐term complications and improve quality of life. Indeed, repeated GIP positivity for several days has been reported to be related to intestinal mucosal damage [15, 36]. In the study by Moreno et al., analysis of duodenal biopsies revealed that most patients with CD (89%) who had no villous atrophy showed no detectable GIP in urine, while all patients with quantifiable GIP in urine had an incomplete recovery of the intestinal mucosa [15]. Although GIP testing was conducted using urine in this study, a similar strong correlation likely exists for faecal GIP, which may be even more specific. It has also been demonstrated that urinary GIP tests can be overly sensitiveand may result positive even in subjects perfectly complying with the requirements of the standard GFD [37]. Stool GIP can be detected up to 3–4 days after gluten ingestion, while urine GIP is typically detectable for only 12–24 h, with peak levels at 6–12 h. Fo this reason, stool tests are more likely to catch occasional or accidental gluten intake, especially when testing is not done immediately after exposure. Moreover, some studies suggest that stool GIP reflects intestinal exposure more directly than urine, which may make it a more reliable indicator of mucosal gluten contact [35].

Given the high sensitivity of GIP, it may be useful to perform endoscopy in patients who repeatedly test positive for GIP, in order to detect intestinal damage caused by prolonged ingestion of even minimal quantities of gluten. Additionally, GIP testing can be valuable in symptomatic patients to assess possible gluten contamination and help with self‐management.

We acknowledge that our study has some limitations. First, the small sample size and the evaluation of faecal GIP only on a single stool sample. However, since faecal analysis of GIP is an accurate method for assessing gluten exposure shortly after ingestion, the single stool sample collected in our study may have underestimated GIP positivity. Additionally, this is asingle center study which limits its external validity and precludes the generalisation of its results. As a further limitation of our study, we must underline the lack of a symptomatic score and a dietary recall (e.g., the 3‐day food diary) to evaluate gluten ingestion. Nevertheless, this is one of the few studies conducted exclusively in paediatric patients, confirming the role of faecal GIP as a highly sensitive and minimally invasive method for evaluating gluten exposure. It allows for the identification of non‐adherent patients who might otherwise go undetected and suggests that it can be used with refractory CD as an additional tool to determine whether to proceed with a new endoscopy.

In conclusion, the inability to directly measure GFD adherence is an unsolved issue for both clinicians and researchers. GIP testing reveals a low adherence rate among patients on a GFD and highlights the limitations of dietary questionnaires or serological methods for monitoring diet. Therefore, in accordance with the recent ESPGHAN position paper on the management of children with CD, we conclude that GIP can be considered a valuable tool to monitor gluten exposure. However, further multicenter studies with larger cohorts are needed to assess the dose/effect relationship between the quantity of ingested gluten and the amount of urinary/faecal GIP, the latency between gluten exposure and its appearance in stool/urine, and the role of these tests in the assessment of long‐term adherence to GFD.

## Author Contributions

**Sabrina Cenni:** conceptualization, data curation, writing – original draft, investigation. **Marianna Casertano:** conceptualization, investigation, writing – original draft, data curation. **Elisabetta D'Addio:** investigation. **Daniela Pacella:** data curation, writing – original draft. **Carlo Tolone:** resources, conceptualization, writing – review and editing. **Massimo Martinelli:** conceptualization, resources, writing – review and editing. **Annamaria Staiano:** conceptualization, writing – review and editing, resources. **Erasmo Miele:** resources, writing – review and editing, conceptualization. **Caterina Strisciuglio:** conceptualization, writing – review and editing, resources, supervision.

## Ethics Statement

The Institutional Review Board of the University of Campania ‘Luigi Vanvitelli’ approved the study protocol with the registration number 207/20.

## Conflicts of Interest

The authors declare no conflicts of interest.

## Data Availability

Our data are entered into a database and are available by agreement with authors.
